# A two-dimensional coordination polymer: poly[[bis­[μ_2_-*N*-ethyl-*N*-(pyridin-4-ylmeth­yl)di­thio­carbamato-κ^3^ *N*:*S*,*S*′]cadmium(II)] 3-methyl­pyridine monosolvate]

**DOI:** 10.1107/S2056989017003516

**Published:** 2017-03-10

**Authors:** Hadi D. Arman, Pavel Poplaukhin, Edward R. T. Tiekink

**Affiliations:** a Department of Chemistry, The University of Texas at San Antonio, One UTSA Circle, San Antonio, Texas 78249-0698, USA; b Chemical Abstracts Service, 2540 Olentangy River Rd, Columbus, Ohio 43202, USA; c Centre for Crystalline Materials, School of Science and Technology, Sunway University, 47500 Bandar Sunway, Selangor Darul Ehsan, Malaysia

**Keywords:** crystal structure, coordination polymer, cadmium, di­thio­carbamate, hy­droxy­pyridine

## Abstract

The title compound, {[Cd(C_9_H_11_N_2_S_2_)_2_]·C_6_H_7_N}_*n*_, features two μ_2_-κ^3^-di­thio­carbamate ligands each of which chelates one Cd^II^ atom, *via* the S atoms, while simultaneously bridging to another *via* the pyridyl-N atom. The result is a two-dimensional coordination polymer extending parallel to the *ab* plane with square channels along the *b* axis. The Cd^II^ atom geometry is based on a distorted *cis*-N_2_S_4_ octa­hedron. The 3-methyl­pyridine mol­ecules reside in the channels aligned along the *b* axis, being held in place by methyl­ene-C—H⋯N(3-methyl­pyridine) and (3-methyl­pyridine)-C—H⋯π(pyrid­yl) inter­actions. Pyridyl-C—H⋯S and di­thio­carbamate-methyl-C—H⋯π(pyrid­yl) inter­actions provide connections between layers along the *c* axis.

## Abstract 2

The title compound, {Cd[S_2_CN(Et)CH_2_py]_2_.3-methyl­pyridine}_*n*_, is a two-dimensional coordination polymer with square channels in which reside the 3-methyl­pyridine mol­ecules.

## Chemical context   

Despite the relatively recent observations of one-dimensional coordination polymers for some binary cadmium di­thio­carbamates (Tan *et al.*, 2013 ▸, 2016 ▸; Ferreira *et al.*, 2016 ▸), *i.e*. compounds of general formula Cd(S_2_CN*RR*′)_2_, for *R*, *R*′ = alkyl, aryl, the overwhelming majority of Cd(S_2_CN*RR*′)_2_ structures are binuclear and zero-dimensional (*i.e*. mol­ecular). This arises owing to the presence of equal numbers of chelating ligands and tridentate ligands, with the latter chelating one Cd^II^ atom while bridging a second. The coord­ination geometry defined by the resulting S_5_ donor set is invariably highly distorted and inter­mediate between trigonal-bipyramidal and square-pyramidal (Tiekink, 2003 ▸). The polymeric motifs of Cd(S_2_CN*RR*′)_2_ have μ_3_-bridging ligands exclusively and six-coordinate, S_6_, geometries. Systematic crystallization studies indicate these transform to the binuclear motif with the egress of time (Tan *et al.*, 2013 ▸, 2016 ▸), suggesting the zero-dimensional motif is the thermodynamic outcome of crystallization. The addition of monodentate pyridyl-N donor mol­ecules during adduct formation more often than not results in the breakdown of the binuclear motif to form a mononuclear species, *e.g*. as in the structures of Cd{S_2_CN[CH_2_C(H)Me_2_]_2_}_2_(pyridine) (Rodina *et al.*, 2011 ▸) and Cd[S_2_CN(Me)Ph]_2_(pyridine)_2_ (Onwudiwe *et al.*, 2013 ▸). The latter structure shows it is possible for the Cd^II^ atom to increase its coordination number to six in the presence of N-donors. Hence, bipyridyl donors with suitably disposed nitro­gen atoms might be anti­cipated to produce coordination polymers. This has been realized in several examples, *e.g*. in the one-dimensional coordination polymers of {Cd(S_2_CNEt_2_)_2_[1,2-bis­(pyridin-4-yl)ethyl­ene]}_*n*_ (Chai *et al.*, 2003 ▸) and in its 1,2-bis­(pyridin-4-yl)ethane analogue (Avila *et al.*, 2006 ▸). In these instances, the Cd^II^ atom exists within a *trans*-N_2_S_4_ coordination geometry. However, the reaction outcomes are not always as expected.
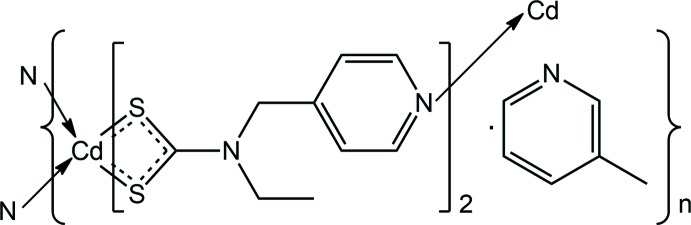


Thus, in [{Cd[S_2_C(*i*Pr)CH_2_CH_2_OH]_2_}_2_[1,2-bis­(pyridin-4-yl)ethyl­ene]_3_], isolated as its tetra aceto­nitrile solvate, both bidentate bridging (× 1) and monodentate (× 2) modes of coordination are found for 1,2-bis­(pyridin-4-yl)ethyl­ene, resulting in a *cis*-N_2_S_4_ coordination geometry (Jotani, Poplaukhin, *et al.*, 2016 ▸). In another unexpected reaction outcome, only monodentate modes of coordination are found for 3-pyridine­aldazine in the structure of {Cd[S_2_CN(*n*Pr)CH_2_CH_2_OH]_2_(3-pyridine­aldazine)_2_} leading to a NS_4_ donor set for cadmium (Broker & Tiekink, 2011 ▸). Very recently, a more surprising structure was reported wherein the binuclear core usually found for Cd(S_2_CN*RR*′)_2_, see above, was retained. Thus, the structure of {Cd[S_2_CN(*i*Pr)CH_2_CH_2_OH]_2_(3-pyridine­aldazine)}_2_, isolated as its hydrate (Arman *et al.*, 2016 ▸), features monodentate binding of the 3-pyridine­aldazine ligands to each Cd^II^ atom, leading to NS_5_ coordination geometries.

The varied and inter­esting structures notwithstanding, it is obvious that Cd^II^ will expand its coordination number in the presence of pyridyl-N donors. Hence, in order to encourage the formation of higher-dimensional aggregates, functionalizing the di­thio­carbamate ligand with pyridyl substituents offers an opportunity to increase the dimensionality of the structure. Indeed, Cd^II^ structures with pyridin-4-yl groups included in the di­thio­carbamate ligand have appeared in the recent literature, *e.g*. Cd[S_2_CN(ferrocenylmeth­yl)CH_2_Py]_2_(1,10-phenanthroline) (Kumar *et al.*, 2016 ▸). Here, the Cd^II^ atom is coordinatively saturated within a *cis*-N_2_S_4_ donor set so the pyridyl-N atoms of the di­thio­carbamate ligand are non-coordinating. However, pyridyl-N bridging has been observed in the binuclear structure, [Cd[S_2_CN(1*H*-indol-3-ylmeth­yl)CH_2_(CH_2_py)]_2_}_2_ (Kumar *et al.*, 2014 ▸). This structure is in fact very closely related to the common binuclear motif but, instead of a bridging, tridentate di­thio­carbamate ligand, *via* three sulfur donors, the bridges in this structure are provided by the pyridyl-N atoms; the two pendent pyridyl groups are non-coordinating. In a continuation of exploratory work in this field (Arman *et al.*, 2013 ▸), herein the crystal and mol­ecular structures of the title two-dimensional coordination polymer, (I), {Cd[S_2_CN(Et)CH_2_py]2.3-methyl­pyridine}_2_, containing a pyridyl-functionalized di­thio­carbamate ligand, is described.

## Structural commentary   

**Figure 1:**
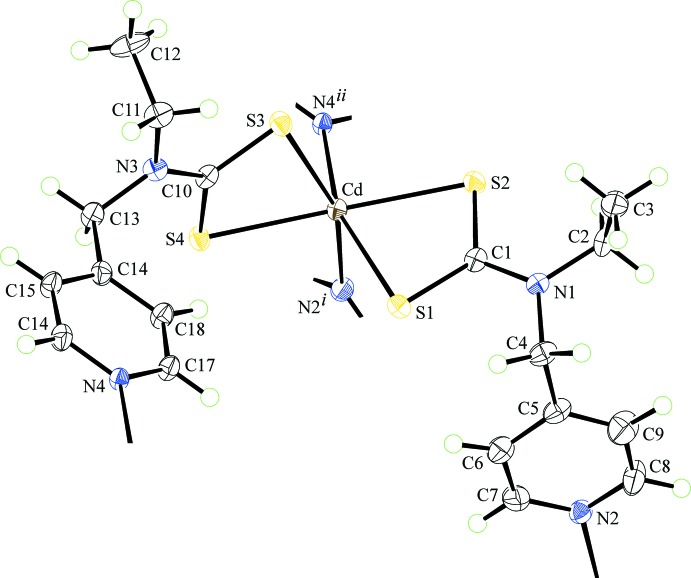
The Cd[S_2_CN(Et)CH_2_py]_2_ component of the asymmetric unit of (I), extended to show the immediate coordination geometry about the Cd^II^ atom, showing the atom-labelling scheme and displacement ellipsoids at the 50% probability level. [Symmetry codes: (i) −*x*, −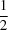 + *y*, −*z*; (ii) −1 + *x*, *y*, *z*.]

**Figure 2:**
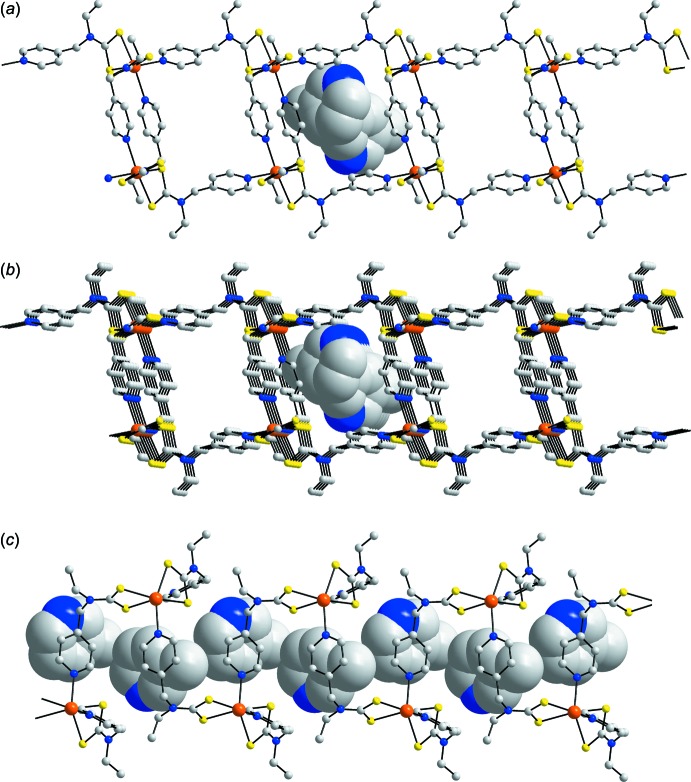
The two-dimensional architecture in (I), showing (*a*) a view in projection down the *a* axis, (*b*) a view slightly off-set from the *a* axis and (*c*) a view in projection down the *b* axis. The 3-methyl­pyridine mol­ecules are shown in space-filling mode. All H atoms have been removed for reasons of clarity.

The asymmetric unit of (I) comprises a molecule of Cd[S_2_CN(Et)CH_2_py]_2_, Fig. 1 ▸, and a mol­ecule of 3-methyl­pyridine. Referring to Table 1 ▸, each di­thio­carbamate anion is chelating, forming very similar Cd—S bond lengths. This similarity is reflected in the experimental equivalence of the associated C—S bond lengths. Each di­thio­carbamate ligand is in fact tridentate, chelating one Cd^II^ atom as just described and simultaneously bridging another *via* the pyridyl-N atom so that the coord­ination geometry about the Cd^II^ atom is *cis*-N_2_S_4_, distorted octa­hedral, Table 1 ▸. The bridging extends to form two inter­connected rows of mol­ecules, with those aligned along the *a* axis being formed *via* S3/S4–N4 bridges and those along the *b* axis being sustained by S1/S2–N2 bridges. The result is a two-dimensional architecture in the *ab* plane, Fig. 2 ▸. Square channels are formed in the *b*-axis direction and these are occupied by the solvent 3-methyl­pyridine mol­ecules, Fig. 2 ▸ *a* and *b*. The slats along the *a* axis are defined by the pyridyl residues and these block access along this direction, Fig. 2 ▸ *c*.

## Supra­molecular features   

**Figure 3:**
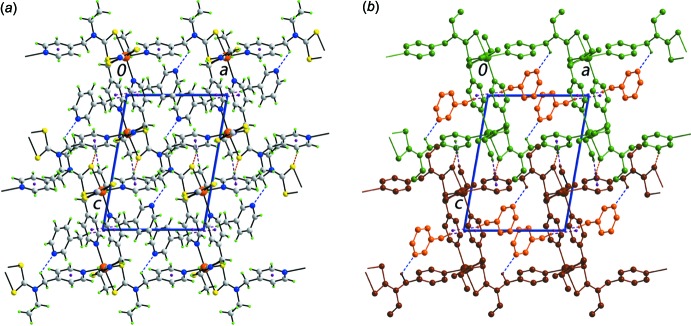
Two representations of the mol­ecular packing in (I), showing (*a*) a view of the unit-cell contents in projection down the *b* axis and (*b*) a simplified view where all H atoms not participating in the specified inter­molecular contacts are removed, adjacent layers are coloured in green and brown, and 3-methyl­pyridine mol­ecules are coloured orange. The C—H⋯S, C—H⋯N and C—H⋯π inter­actions are shown as orange, blue and purple dashed lines, respectively.

A summary of specific inter­molecular inter­actions contributing to the mol­ecular packing of (I) is given in Table 2 ▸. The main inter­actions between the host framework and the guest 3-methyl­pyridine mol­ecules are of the type methyl­ene-C—H⋯N(3-methyl­pyridine) and (3-methyl­pyridine)-C—H⋯π(pyrid­yl). The connections between layers stacking along the *c* axis are of the type pyridyl-C—H⋯S and di­thio­carbamate-methyl-C—H⋯π(pyrid­yl). Two illustrations of the mol­ecular packing are given in Fig. 3 ▸.

## Database survey   

The dithiocarbamate anion, ^−^[S_2_CN(Et)CH_2_py], found in (I) has been reported in a series of diorganotin bis­(di­thio­carbamate)s (Barba *et al.*, 2012 ▸) but there was no evidence for inter­molecular Sn—N(py) inter­actions, the structures rather conforming to the expected motifs (Tiekink, 2008 ▸). There are also examples of structures of the general formula *M*[S_2_CN(*R*)CH_2_py]_2_, a notable example being one with *R* = CH_2_py, namely, {Hg[S_2_CN(CH_2_Py)_2_]_2_]}_*n*_ (Yadav *et al.*, 2014 ▸), *i.e*. with two pyridyl groups per di­thio­carbamate ligand, which adopts a relatively rare one-dimensional coordination polymer with a twisted topology (Jotani, Tan *et al.*, 2016 ▸). In the other structures, *R* is a non-coordinating residue. For example, in the centrosymmetric Zn^II^ compound with *R* = CH_2_(ferrocen­yl) (Kumar *et al.*, 2016 ▸), a two-dimensional architecture is found. Reverting back to Hg^II^ structures, when *R* = CH_2_(fur­yl) (Kumar *et al.*, 2016 ▸), a flat, two-dimensional architecture is found as the Hg^II^ atom lies on a centre on inversion. In the case of {Hg[S_2_CN(Me)CH_2_Py]_2_}_*n*_ (Singh *et al.*, 2014 ▸), mol­ecules self-assemble into a one-dimensional coordination polymer as one pyridyl-N atom coordinates a neighbouring Hg^II^ atom while the other is non-coordinating. Finally, when *R* = CH_2_(1-methyl-1*H*-pyrrol-2-yl) (Yadav *et al.*, 2014 ▸), no Hg—N inter­actions are found. The Hg^II^ atom has a distorted tetra­hedral geometry defined by an S_4_ donor set. Such a variety in structures warrants continuing inter­est in this area.

## Synthesis and crystallization   

The Cd[S_2_CN(Et_2_)CH_2_py]_2_ precursor (268 mg, 0.50 mmol) was dissolved in an excess of 3-methyl­pyridine (*ca* 10 ml). The solution was filtered, transferred to a 50 ml test tube and layered with hexa­nes (*ca* 60 ml). Colourless crystals of (I) formed on the test tube walls within a week. IR (cm^−1^): 2973(*w*), 2923(*w*), 1608(*s*), 1473(*s*), 1408(*s*), 1283(*m*), 1249(*m*), 1219(*s*), 1167(*s*), 1107(*m*), 1071(*m*), 991(*s*), 946(*s*). NMR ^1^H: δ (ppm) 8.56 (*dd*, Ar, 2.98, 5.49 Hz), 8.43 (*t*, Ar, 1.00 Hz), 8.38 (*dd*, Ar, 0.89, 3.88 Hz), 7.61 (*dq*, Ar, 1.49, 7.82 Hz), 7.30 (*d*, Ar, 6.02 Hz), 5.19 (*s*, –CH_2_–Ar), 3.88 (*q*, –C*H*_2_CH_3_, 6.49 Hz), 2.30 (*s*, pyridyl-CH_3_), 1.22 (*t*, –C*H*_2_CH_3_, 4.80 Hz). M.p. 531 – 533 K (uncorrected). TGA: two steps, the first corresponding to loss of 3-methyl­pyridine (onset 410 K, mid-point 420 K, endset 431 K; theoretical mass loss 14.8%, observed mass loss 13.3%), the second step corresponds to the decomposition to CdS (onset 603 K, mid-point 604 K, endset 613 K; theoretical mass loss 62.3%, observed mass loss 57.4%). Total theoretical mass loss 77.1%, observed mass loss 74.9%.

## Refinement details   

Crystal data, data collection and structure refinement details are summarized in Table 3 ▸. The carbon-bound H atoms were placed in calculated positions (C—H = 0.95–0.99 Å) and were included in the refinement in the riding-model approximation, with *U*_iso_(H) set to 1.2–1.5*U*_eq_(C). Owing to inter­ference from the beam-stop, the (100) reflection was removed from the final cycles of refinement.

## Supplementary Material

Crystal structure: contains datablock(s) I, global. DOI: 10.1107/S2056989017003516/hb7664sup1.cif

Structure factors: contains datablock(s) I. DOI: 10.1107/S2056989017003516/hb7664Isup2.hkl

CCDC reference: 1535967

Additional supporting information:  crystallographic information; 3D view; checkCIF report

## Figures and Tables

**Table 1:** Selected geometric parameters (Å, °)

Cd—S1	2.6399 (19)	Cd—N4^ii^	2.346 (5)
Cd—S2	2.6618 (17)	S1—C1	1.714 (6)
Cd—S3	2.6578 (16)	S2—C1	1.715 (6)
Cd—S4	2.6932 (18)	S3—C10	1.715 (6)
Cd—N2^i^	2.430 (5)	S4—C10	1.724 (6)
			
S1—Cd—S2	68.26 (5)	S2—Cd—N4^ii^	94.15 (14)
S1—Cd—S3	101.88 (6)	S3—Cd—S4	67.58 (5)
S1—Cd—S4	100.71 (5)	S3—Cd—N2^i^	158.55 (18)
S1—Cd—N2^i^	90.18 (16)	S3—Cd—N4^ii^	91.47 (13)
S1—Cd—N4^ii^	159.47 (13)	S4—Cd—N2^i^	92.95 (16)
S2—Cd—S3	100.73 (5)	S4—Cd—N4^ii^	98.77 (14)
S2—Cd—S4	162.67 (5)	N2^i^—Cd—N4^ii^	82.40 (18)
S2—Cd—N2^i^	100.20 (17)		

Symmetry codes: (i) 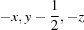; (ii) 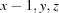.

**Table 2:** Hydrogen-bond geometry (Å, °) *Cg*1 and *Cg*2 are the centroids of the N2/C5–C9 and N4/C14–C17 rings, respectively.

*D*—H⋯*A*	*D*—H	H⋯*A*	*D*⋯*A*	*D*—H⋯*A*
C13—H13*A*⋯N5^iii^	0.99	2.62	3.264 (15)	123
C24—H24*C*⋯*Cg*1^iv^	0.98	2.72	3.662 (12)	163
C15—H15⋯S3^iii^	0.95	2.81	3.738 (7)	167
C3—H3*C*⋯*Cg*2^v^	0.98	2.73	3.633 (8)	154

Symmetry codes: (iii) 
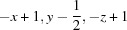
; (iv) 
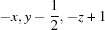
; (v) 
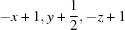
.

**Table 3:** Experimental details

Crystal data	
Chemical formula	[Cd(C_9_H_11_N_2_S_2_)_2_]·C_6_H_7_N
*M*_r_	628.16
Crystal system, space group	Monoclinic, *P*2_1_
Temperature (K)	98
*a*, *b*, *c* (Å)	9.5842 (15), 11.0788 (16), 12.989 (2)
β (°)	100.014 (4)
*V* (Å^3^)	1358.2 (4)
*Z*	2
Radiation type	Mo *K*α
μ (mm^−1^)	1.13
Crystal size (mm)	0.23 × 0.20 × 0.10

Data collection	
Diffractometer	AFC12/SATURN724
Absorption correction	Multi-scan (*ABSCOR*; Higashi, 1995 ▸)
*T*_min_, *T*_max_	0.802, 1.000
No. of measured, independent and observed [*I* > 2σ(*I*)] reflections	10508, 5618, 5586
*R*_int_	0.030
(sin θ/λ)_max_ (Å^−1^)	0.650

Refinement	
*R*[*F* ^2^ > 2σ(*F* ^2^)], *wR*(*F* ^2^), *S*	0.037, 0.091, 1.08
No. of reflections	5618
No. of parameters	310
No. of restraints	1
H-atom treatment	H-atom parameters constrained
Δρ_max_, Δρ_min_ (e Å^−3^)	0.95, −1.20
Absolute structure	Flack *x* determined using 2244 quotients [(*I* ^+^)−(*I* ^−^)]/[(*I* ^+^)+(*I* ^−^)] (Parsons *et al.*, 2013 ▸)
Absolute structure parameter	0.035 (15)

Computer programs: *CrystalClear* (Molecular Structure Corporation & Rigaku, 2005 ▸), *SHELXS97* (Sheldrick, 2008 ▸), *SHELXL2014* (Sheldrick, 2015 ▸), *ORTEP-3 for Windows* (Farrugia, 2012 ▸), *DIAMOND* (Brandenburg, 2006 ▸) and *publCIF* (Westrip, 2010 ▸).

## supplementary crystallographic information

### Crystal data

**Table:**

[Cd(C_9_H_11_N_2_S_2_)_2_]·C_6_H_7_N	*F*(000) = 640
*M**_r_* = 628.16	*D*_x_ = 1.536 Mg m^−^^3^
Monoclinic, *P*2_1_	Mo *K*α radiation, λ = 0.71069 Å
*a* = 9.5842 (15) Å	Cell parameters from 6543 reflections
*b* = 11.0788 (16) Å	θ = 2.4–40.7°
*c* = 12.989 (2) Å	µ = 1.13 mm^−^^1^
β = 100.014 (4)°	*T* = 98 K
*V* = 1358.2 (4) Å^3^	Block, colourless
*Z* = 2	0.23 × 0.20 × 0.10 mm

### Data collection

**Table:**

AFC12K/SATURN724 diffractometer	5618 independent reflections
Radiation source: fine-focus sealed tube	5586 reflections with *I* > 2σ(*I*)
Graphite monochromator	*R*_int_ = 0.030
ω scans	θ_max_ = 27.5°, θ_min_ = 2.4°
Absorption correction: multi-scan (ABSCOR; Higashi, 1995)	*h* = −12→12
*T*_min_ = 0.802, *T*_max_ = 1.000	*k* = −14→13
10508 measured reflections	*l* = −15→16

### Refinement

**Table:**

Refinement on *F*^2^	Hydrogen site location: inferred from neighbouring sites
Least-squares matrix: full	H-atom parameters constrained
*R*[*F*^2^ > 2σ(*F*^2^)] = 0.037	*w* = 1/[σ^2^(*F*_o_^2^) + (0.0304*P*)^2^ + 3.8335*P*] where *P* = (*F*_o_^2^ + 2*F*_c_^2^)/3
*wR*(*F*^2^) = 0.091	(Δ/σ)_max_ < 0.001
*S* = 1.08	Δρ_max_ = 0.95 e Å^−^^3^
5618 reflections	Δρ_min_ = −1.20 e Å^−^^3^
310 parameters	Absolute structure: Flack *x* determined using 2244 quotients [(*I*^+^)-(*I*^-^)]/[(*I*^+^)+(*I*^-^)] (Parsons *et al.*, 2013)
1 restraint	Absolute structure parameter: 0.035 (15)

### Special details

**Table:**

Geometry. All esds (except the esd in the dihedral angle between two l.s. planes) are estimated using the full covariance matrix. The cell esds are taken into account individually in the estimation of esds in distances, angles and torsion angles; correlations between esds in cell parameters are only used when they are defined by crystal symmetry. An approximate (isotropic) treatment of cell esds is used for estimating esds involving l.s. planes.
Refinement. Being affected by the beamstop, the (100) reflection was omitted from the final cycles of refinement.

### Fractional atomic coordinates and isotropic or equivalent isotropic displacement parameters (Å^2^)

**Table:**

	*x*	*y*	*z*	*U*_iso_*/*U*_eq_	
Cd	0.07792 (4)	0.11775 (5)	0.28476 (3)	0.01706 (11)	
S1	0.23327 (18)	0.29766 (16)	0.23386 (13)	0.0188 (3)	
S2	−0.01802 (17)	0.33065 (15)	0.33697 (13)	0.0208 (3)	
S3	0.22627 (17)	0.04779 (16)	0.46716 (12)	0.0215 (3)	
S4	0.24814 (18)	−0.07121 (16)	0.26516 (13)	0.0198 (3)	
N1	0.1324 (5)	0.5121 (5)	0.2772 (4)	0.0171 (10)	
N2	0.0426 (5)	0.6032 (7)	−0.1044 (4)	0.0216 (12)	
N3	0.4308 (5)	−0.1054 (5)	0.4433 (4)	0.0182 (10)	
N4	0.8778 (5)	0.0113 (5)	0.3160 (4)	0.0163 (10)	
C1	0.1167 (6)	0.3905 (6)	0.2819 (4)	0.0160 (11)	
C2	0.0472 (7)	0.5963 (6)	0.3283 (5)	0.0242 (15)	
H2A	−0.0413	0.5561	0.3394	0.029*	
H2B	0.0213	0.6674	0.2829	0.029*	
C3	0.1312 (8)	0.6364 (8)	0.4323 (5)	0.0316 (18)	
H3A	0.0725	0.6894	0.4675	0.047*	
H3B	0.2159	0.6802	0.4206	0.047*	
H3C	0.1595	0.5655	0.4761	0.047*	
C4	0.2370 (7)	0.5677 (7)	0.2207 (5)	0.0238 (14)	
H4A	0.3221	0.5157	0.2271	0.029*	
H4B	0.2664	0.6472	0.2520	0.029*	
C5	0.1735 (7)	0.5838 (6)	0.1065 (5)	0.0243 (15)	
C6	0.1958 (8)	0.4960 (7)	0.0331 (6)	0.0314 (16)	
H6	0.2563	0.4289	0.0528	0.038*	
C7	0.1261 (8)	0.5106 (7)	−0.0692 (6)	0.0313 (16)	
H7	0.1392	0.4496	−0.1181	0.038*	
C8	0.0322 (9)	0.6904 (7)	−0.0345 (6)	0.0357 (18)	
H8	−0.0213	0.7607	−0.0571	0.043*	
C9	0.0971 (10)	0.6820 (8)	0.0704 (6)	0.0378 (19)	
H9	0.0872	0.7463	0.1169	0.045*	
C10	0.3130 (6)	−0.0482 (6)	0.3960 (5)	0.0158 (11)	
C11	0.4862 (6)	−0.0914 (7)	0.5560 (5)	0.0239 (14)	
H11A	0.4691	−0.0078	0.5778	0.029*	
H11B	0.5898	−0.1056	0.5694	0.029*	
C12	0.4151 (8)	−0.1797 (9)	0.6200 (6)	0.0344 (19)	
H12A	0.4554	−0.1706	0.6942	0.052*	
H12B	0.4308	−0.2624	0.5977	0.052*	
H12C	0.3131	−0.1632	0.6092	0.052*	
C13	0.5156 (6)	−0.1836 (6)	0.3869 (5)	0.0216 (13)	
H13A	0.4562	−0.2126	0.3216	0.026*	
H13B	0.5485	−0.2548	0.4305	0.026*	
C14	0.6427 (7)	−0.1156 (6)	0.3609 (5)	0.0216 (13)	
C15	0.7786 (6)	−0.1612 (6)	0.3879 (5)	0.0225 (13)	
H15	0.7938	−0.2369	0.4224	0.027*	
C16	0.8927 (7)	−0.0960 (6)	0.3645 (5)	0.0243 (14)	
H16	0.9852	−0.1288	0.3836	0.029*	
C17	0.7450 (7)	0.0563 (6)	0.2896 (5)	0.0214 (13)	
H17	0.7326	0.1319	0.2547	0.026*	
C18	0.6265 (7)	−0.0034 (6)	0.3115 (5)	0.0218 (13)	
H18	0.5351	0.0318	0.2930	0.026*	
N5	0.4417 (12)	0.1672 (11)	0.8221 (11)	0.084 (4)	
C19	0.3520 (10)	0.0783 (11)	0.8364 (9)	0.059 (3)	
H19	0.3143	0.0284	0.7787	0.071*	
C20	0.3115 (11)	0.0565 (10)	0.9343 (8)	0.052 (2)	
C21	0.3647 (10)	0.1275 (16)	1.0165 (8)	0.060 (3)	
H21	0.3389	0.1126	1.0827	0.072*	
C22	0.4575 (14)	0.2230 (14)	1.0056 (12)	0.081 (4)	
H22	0.4972	0.2730	1.0627	0.097*	
C23	0.4878 (15)	0.2396 (15)	0.9037 (12)	0.081 (4)	
H23	0.5448	0.3068	0.8923	0.097*	
C24	0.2137 (11)	−0.0448 (12)	0.9431 (9)	0.063 (3)	
H24A	0.2640	−0.1078	0.9879	0.094*	
H24B	0.1789	−0.0782	0.8735	0.094*	
H24C	0.1335	−0.0156	0.9736	0.094*	

### Atomic displacement parameters (Å^2^)

**Table:**

	*U*^11^	*U*^22^	*U*^33^	*U*^12^	*U*^13^	*U*^23^
Cd	0.01767 (18)	0.01518 (19)	0.01811 (19)	0.0001 (2)	0.00250 (13)	0.00003 (19)
S1	0.0144 (7)	0.0213 (9)	0.0207 (7)	−0.0016 (6)	0.0030 (6)	−0.0033 (6)
S2	0.0208 (7)	0.0181 (8)	0.0255 (8)	−0.0002 (6)	0.0095 (6)	−0.0005 (6)
S3	0.0231 (8)	0.0224 (9)	0.0183 (7)	0.0048 (6)	0.0016 (6)	−0.0022 (6)
S4	0.0218 (8)	0.0182 (9)	0.0187 (7)	0.0025 (6)	0.0021 (6)	−0.0031 (6)
N1	0.017 (2)	0.018 (3)	0.016 (2)	−0.002 (2)	0.0005 (19)	0.0004 (19)
N2	0.027 (2)	0.019 (3)	0.019 (2)	0.000 (2)	0.0026 (18)	0.000 (2)
N3	0.010 (2)	0.022 (3)	0.022 (3)	−0.003 (2)	0.0009 (18)	0.000 (2)
N4	0.014 (2)	0.015 (3)	0.021 (2)	0.0000 (19)	0.0028 (18)	0.0007 (19)
C1	0.011 (3)	0.020 (3)	0.013 (3)	−0.001 (2)	−0.0073 (19)	0.000 (2)
C2	0.027 (3)	0.015 (4)	0.030 (3)	0.003 (2)	0.002 (2)	0.002 (2)
C3	0.046 (4)	0.028 (5)	0.020 (3)	0.004 (3)	0.005 (3)	−0.001 (3)
C4	0.024 (3)	0.023 (3)	0.024 (3)	−0.009 (3)	0.001 (2)	0.002 (2)
C5	0.029 (3)	0.022 (4)	0.020 (3)	−0.007 (2)	0.000 (3)	0.002 (2)
C6	0.035 (4)	0.032 (4)	0.027 (4)	0.008 (3)	0.005 (3)	0.008 (3)
C7	0.038 (4)	0.034 (4)	0.023 (3)	0.008 (3)	0.008 (3)	0.003 (3)
C8	0.051 (5)	0.021 (4)	0.031 (4)	0.009 (3)	−0.004 (3)	0.000 (3)
C9	0.055 (5)	0.028 (5)	0.027 (4)	−0.006 (4)	−0.004 (3)	−0.007 (3)
C10	0.008 (2)	0.017 (3)	0.020 (3)	−0.004 (2)	−0.003 (2)	0.002 (2)
C11	0.011 (3)	0.032 (4)	0.027 (3)	−0.003 (3)	−0.002 (2)	0.005 (3)
C12	0.026 (4)	0.050 (6)	0.024 (3)	−0.012 (4)	−0.004 (3)	0.016 (3)
C13	0.014 (3)	0.020 (3)	0.031 (3)	0.000 (2)	0.004 (2)	0.004 (3)
C14	0.024 (3)	0.016 (3)	0.026 (3)	0.001 (3)	0.007 (3)	−0.001 (2)
C15	0.016 (3)	0.018 (3)	0.035 (4)	0.000 (2)	0.007 (2)	0.006 (3)
C16	0.024 (3)	0.021 (3)	0.028 (3)	0.006 (3)	0.004 (3)	0.003 (3)
C17	0.020 (3)	0.018 (3)	0.026 (3)	0.002 (2)	0.003 (2)	0.004 (3)
C18	0.017 (3)	0.020 (3)	0.028 (3)	0.000 (2)	0.002 (2)	0.004 (3)
N5	0.068 (7)	0.083 (9)	0.114 (10)	0.023 (6)	0.054 (7)	0.041 (7)
C19	0.038 (5)	0.082 (10)	0.061 (6)	0.014 (5)	0.018 (4)	0.014 (5)
C20	0.051 (6)	0.052 (6)	0.059 (6)	0.010 (5)	0.023 (5)	0.001 (5)
C21	0.055 (5)	0.076 (8)	0.053 (5)	−0.006 (7)	0.021 (4)	−0.009 (7)
C22	0.066 (8)	0.078 (10)	0.109 (11)	0.009 (7)	0.042 (8)	0.008 (8)
C23	0.071 (9)	0.081 (10)	0.100 (11)	0.005 (7)	0.042 (8)	0.025 (8)
C24	0.047 (6)	0.074 (8)	0.067 (7)	−0.003 (5)	0.010 (5)	−0.001 (6)

### Geometric parameters (Å, º)

**Table:**

Cd—S1	2.6399 (19)	C7—H7	0.9500
Cd—S2	2.6618 (17)	C8—C9	1.398 (11)
Cd—S3	2.6578 (16)	C8—H8	0.9500
Cd—S4	2.6932 (18)	C9—H9	0.9500
Cd—N2^i^	2.430 (5)	C11—C12	1.520 (9)
Cd—N4^ii^	2.346 (5)	C11—H11A	0.9900
S1—C1	1.714 (6)	C11—H11B	0.9900
S2—C1	1.715 (6)	C12—H12A	0.9800
S3—C10	1.715 (6)	C12—H12B	0.9800
S4—C10	1.724 (6)	C12—H12C	0.9800
N1—C1	1.357 (8)	C13—C14	1.519 (9)
N1—C4	1.477 (8)	C13—H13A	0.9900
N1—C2	1.471 (8)	C13—H13B	0.9900
N2—C8	1.342 (10)	C14—C15	1.385 (9)
N2—C7	1.332 (10)	C14—C18	1.396 (9)
N2—Cd^iii^	2.430 (5)	C15—C16	1.388 (9)
N3—C10	1.347 (8)	C15—H15	0.9500
N3—C13	1.467 (8)	C16—H16	0.9500
N3—C11	1.477 (8)	C17—C18	1.386 (9)
N4—C16	1.341 (9)	C17—H17	0.9500
N4—C17	1.354 (8)	C18—H18	0.9500
N4—Cd^iv^	2.346 (5)	N5—C23	1.341 (19)
C2—C3	1.514 (9)	N5—C19	1.341 (15)
C2—H2A	0.9900	C19—C20	1.413 (13)
C2—H2B	0.9900	C19—H19	0.9500
C3—H3A	0.9800	C20—C21	1.353 (16)
C3—H3B	0.9800	C20—C24	1.479 (15)
C3—H3C	0.9800	C21—C22	1.40 (2)
C4—C5	1.513 (9)	C21—H21	0.9500
C4—H4A	0.9900	C22—C23	1.415 (18)
C4—H4B	0.9900	C22—H22	0.9500
C5—C9	1.349 (11)	C23—H23	0.9500
C5—C6	1.403 (10)	C24—H24A	0.9800
C6—C7	1.389 (10)	C24—H24B	0.9800
C6—H6	0.9500	C24—H24C	0.9800
			
S1—Cd—S2	68.26 (5)	C9—C8—H8	118.7
S1—Cd—S3	101.88 (6)	C5—C9—C8	121.0 (8)
S1—Cd—S4	100.71 (5)	C5—C9—H9	119.5
S1—Cd—N2^i^	90.18 (16)	C8—C9—H9	119.5
S1—Cd—N4^ii^	159.47 (13)	N3—C10—S3	119.5 (5)
S2—Cd—S3	100.73 (5)	N3—C10—S4	120.6 (5)
S2—Cd—S4	162.67 (5)	S3—C10—S4	119.8 (3)
S2—Cd—N2^i^	100.20 (17)	N3—C11—C12	110.8 (6)
S2—Cd—N4^ii^	94.15 (14)	N3—C11—H11A	109.5
S3—Cd—S4	67.58 (5)	C12—C11—H11A	109.5
S3—Cd—N2^i^	158.55 (18)	N3—C11—H11B	109.5
S3—Cd—N4^ii^	91.47 (13)	C12—C11—H11B	109.5
S4—Cd—N2^i^	92.95 (16)	H11A—C11—H11B	108.1
S4—Cd—N4^ii^	98.77 (14)	C11—C12—H12A	109.5
N2^i^—Cd—N4^ii^	82.40 (18)	C11—C12—H12B	109.5
C1—S1—Cd	86.0 (2)	H12A—C12—H12B	109.5
C1—S2—Cd	85.3 (2)	C11—C12—H12C	109.5
C10—S3—Cd	86.3 (2)	H12A—C12—H12C	109.5
C10—S4—Cd	85.0 (2)	H12B—C12—H12C	109.5
C1—N1—C4	121.8 (5)	N3—C13—C14	110.7 (6)
C1—N1—C2	122.3 (5)	N3—C13—H13A	109.5
C4—N1—C2	115.9 (5)	C14—C13—H13A	109.5
C8—N2—C7	115.6 (6)	N3—C13—H13B	109.5
C8—N2—Cd^iii^	121.7 (5)	C14—C13—H13B	109.5
C7—N2—Cd^iii^	122.6 (5)	H13A—C13—H13B	108.1
C10—N3—C13	123.0 (5)	C15—C14—C18	117.8 (6)
C10—N3—C11	122.1 (5)	C15—C14—C13	121.2 (6)
C13—N3—C11	115.0 (5)	C18—C14—C13	121.0 (6)
C16—N4—C17	117.6 (5)	C14—C15—C16	119.8 (6)
C16—N4—Cd^iv^	120.2 (4)	C14—C15—H15	120.1
C17—N4—Cd^iv^	122.1 (4)	C16—C15—H15	120.1
N1—C1—S1	119.7 (5)	N4—C16—C15	122.7 (6)
N1—C1—S2	120.0 (5)	N4—C16—H16	118.6
S1—C1—S2	120.3 (4)	C15—C16—H16	118.6
N1—C2—C3	109.8 (5)	N4—C17—C18	122.7 (6)
N1—C2—H2A	109.7	N4—C17—H17	118.6
C3—C2—H2A	109.7	C18—C17—H17	118.6
N1—C2—H2B	109.7	C17—C18—C14	119.3 (6)
C3—C2—H2B	109.7	C17—C18—H18	120.3
H2A—C2—H2B	108.2	C14—C18—H18	120.3
C2—C3—H3A	109.5	C23—N5—C19	117.4 (11)
C2—C3—H3B	109.5	N5—C19—C20	122.2 (12)
H3A—C3—H3B	109.5	N5—C19—H19	118.9
C2—C3—H3C	109.5	C20—C19—H19	118.9
H3A—C3—H3C	109.5	C21—C20—C19	119.0 (11)
H3B—C3—H3C	109.5	C21—C20—C24	122.5 (10)
N1—C4—C5	110.1 (5)	C19—C20—C24	118.5 (10)
N1—C4—H4A	109.6	C20—C21—C22	121.2 (11)
C5—C4—H4A	109.6	C20—C21—H21	119.4
N1—C4—H4B	109.6	C22—C21—H21	119.4
C5—C4—H4B	109.6	C23—C22—C21	115.3 (14)
H4A—C4—H4B	108.2	C23—C22—H22	122.3
C9—C5—C6	117.4 (7)	C21—C22—H22	122.3
C9—C5—C4	122.5 (7)	N5—C23—C22	124.6 (14)
C6—C5—C4	120.1 (6)	N5—C23—H23	117.7
C7—C6—C5	117.7 (7)	C22—C23—H23	117.7
C7—C6—H6	121.2	C20—C24—H24A	109.5
C5—C6—H6	121.2	C20—C24—H24B	109.5
N2—C7—C6	125.3 (7)	H24A—C24—H24B	109.5
N2—C7—H7	117.3	C20—C24—H24C	109.5
C6—C7—H7	117.3	H24A—C24—H24C	109.5
N2—C8—C9	122.6 (7)	H24B—C24—H24C	109.5
N2—C8—H8	118.7		
			
C4—N1—C1—S1	7.3 (7)	Cd—S3—C10—N3	168.9 (5)
C2—N1—C1—S1	−172.3 (4)	Cd—S3—C10—S4	−11.5 (3)
C4—N1—C1—S2	−173.4 (4)	Cd—S4—C10—N3	−169.0 (5)
C2—N1—C1—S2	7.0 (8)	Cd—S4—C10—S3	11.3 (3)
Cd—S1—C1—N1	−177.9 (5)	C10—N3—C11—C12	85.9 (8)
Cd—S1—C1—S2	2.8 (3)	C13—N3—C11—C12	−95.6 (7)
Cd—S2—C1—N1	177.9 (5)	C10—N3—C13—C14	98.4 (7)
Cd—S2—C1—S1	−2.8 (3)	C11—N3—C13—C14	−80.2 (7)
C1—N1—C2—C3	98.4 (7)	N3—C13—C14—C15	127.2 (7)
C4—N1—C2—C3	−81.3 (7)	N3—C13—C14—C18	−50.9 (8)
C1—N1—C4—C5	86.5 (7)	C18—C14—C15—C16	−0.7 (10)
C2—N1—C4—C5	−93.9 (6)	C13—C14—C15—C16	−179.0 (6)
N1—C4—C5—C9	86.4 (8)	C17—N4—C16—C15	0.0 (10)
N1—C4—C5—C6	−95.1 (8)	Cd^iv^—N4—C16—C15	178.0 (5)
C9—C5—C6—C7	−6.3 (11)	C14—C15—C16—N4	0.1 (11)
C4—C5—C6—C7	175.2 (7)	C16—N4—C17—C18	0.6 (10)
C8—N2—C7—C6	3.1 (12)	Cd^iv^—N4—C17—C18	−177.3 (5)
Cd^iii^—N2—C7—C6	−178.8 (6)	N4—C17—C18—C14	−1.3 (10)
C5—C6—C7—N2	2.0 (12)	C15—C14—C18—C17	1.3 (10)
C7—N2—C8—C9	−4.0 (12)	C13—C14—C18—C17	179.5 (6)
Cd^iii^—N2—C8—C9	177.9 (6)	C23—N5—C19—C20	−3.1 (17)
C6—C5—C9—C8	5.6 (12)	N5—C19—C20—C21	0.1 (16)
C4—C5—C9—C8	−175.9 (7)	N5—C19—C20—C24	−179.1 (10)
N2—C8—C9—C5	−0.4 (14)	C19—C20—C21—C22	0.8 (19)
C13—N3—C10—S3	−176.5 (5)	C24—C20—C21—C22	180.0 (12)
C11—N3—C10—S3	2.0 (8)	C20—C21—C22—C23	1 (2)
C13—N3—C10—S4	3.9 (8)	C19—N5—C23—C22	5 (2)
C11—N3—C10—S4	−177.7 (5)	C21—C22—C23—N5	−4 (2)

Symmetry codes: (i) −*x*, *y*−1/2, −*z*; (ii) *x*−1, *y*, *z*; (iii) −*x*, *y*+1/2, −*z*; (iv) *x*+1, *y*, *z*.

### Hydrogen-bond geometry (Å, º)

*Cg*1 and *Cg*2 are the centroids of the N2/C5–C9 and N4/C14–C17 rings, respectively.

**Table:**

*D*—H···*A*	*D*—H	H···*A*	*D*···*A*	*D*—H···*A*
C13—H13*A*···N5^v^	0.99	2.62	3.264 (15)	123
C24—H24*C*···*Cg*1^vi^	0.98	2.72	3.662 (12)	163
C15—H15···S3^v^	0.95	2.81	3.738 (7)	167
C3—H3*C*···*Cg*2^vii^	0.98	2.73	3.633 (8)	154

Symmetry codes: (v) −*x*+1, *y*−1/2, −*z*+1; (vi) −*x*, *y*−1/2, −*z*+1; (vii) −*x*+1, *y*+1/2, −*z*+1.
